# Supplementary figures and images for: Agonists of melatonin receptors strongly promote the functional recovery from the neuroparalysis induced by neurotoxic snakes
Source: PLoS Negl Trop Dis. 2024 Jan 8;18(1):e0011825. doi: 10.1371/journal.pntd.0011825 (PMC10798625; doi:10.1371/journal.pntd.0011825)

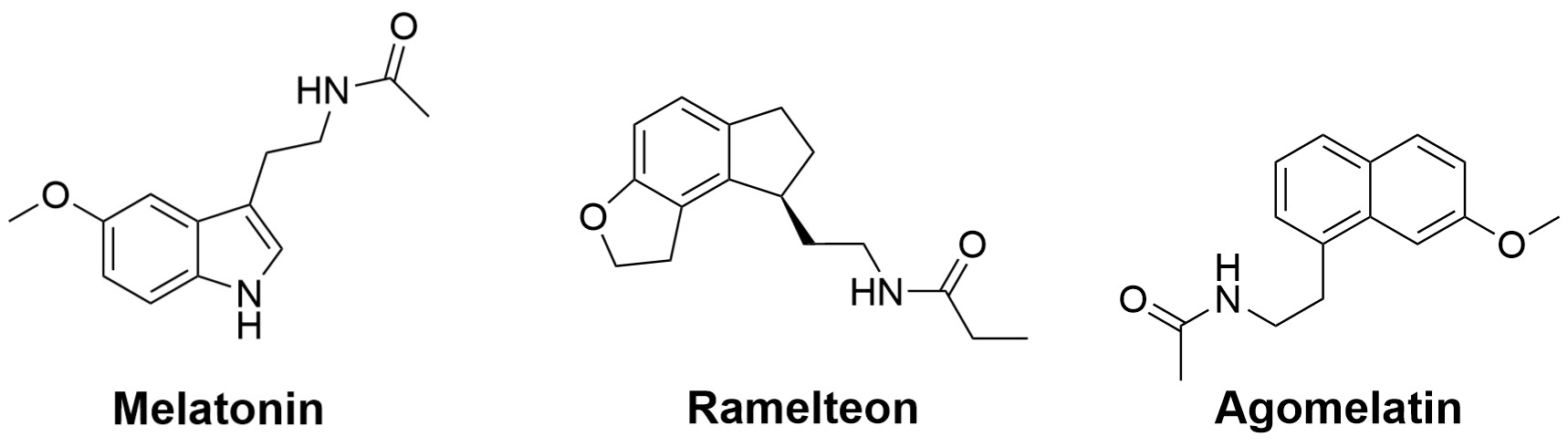

Supplement: S1 Fig — (TIF) [file pntd.0011825.s001.tif]

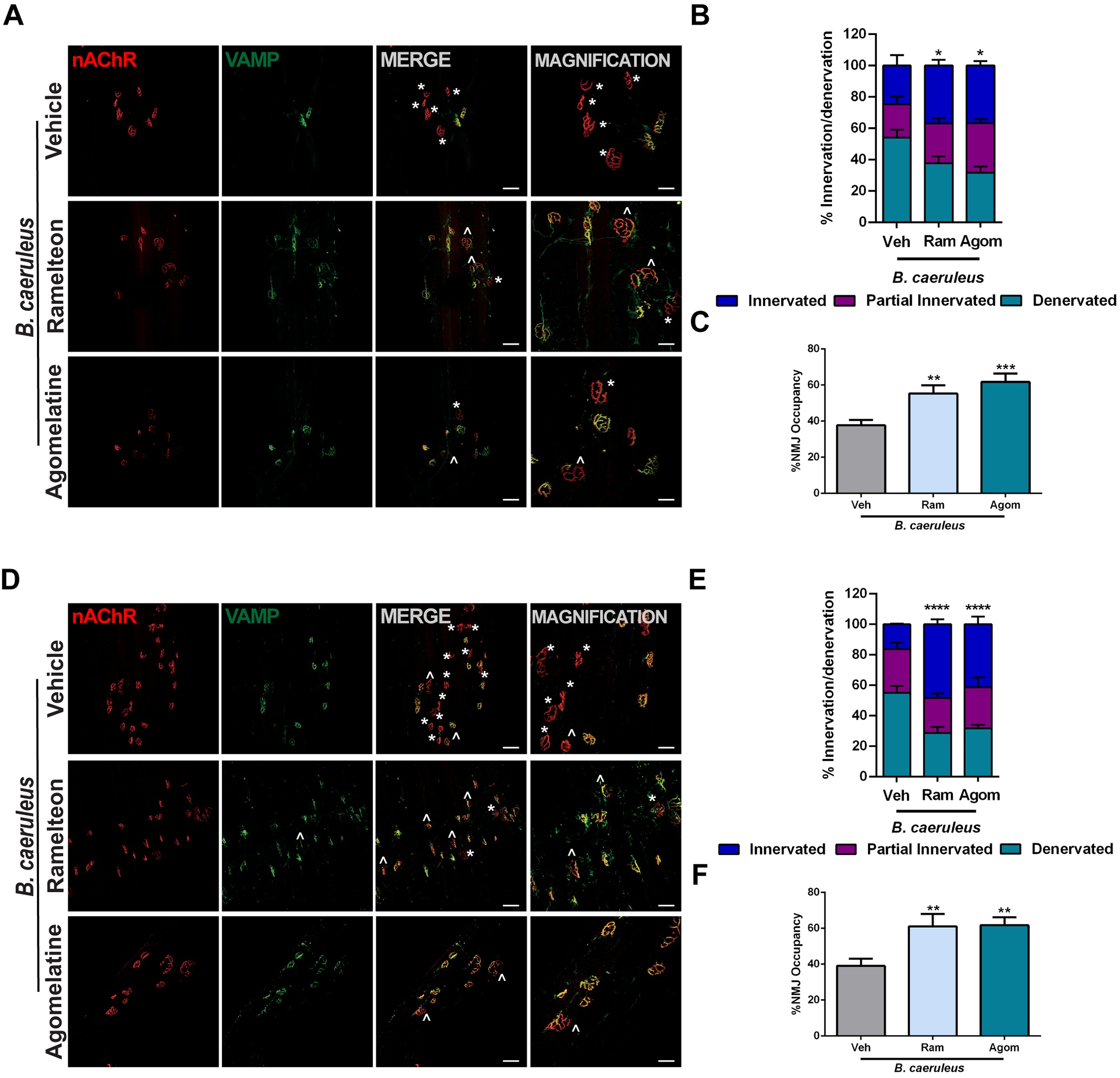

Supplement: S2 Fig — Pectoral (A) and intercostal (D) muscles were processed for indirect immunofluorescence using fluorescent α-BTx to stain post-synaptic AChRs (red), and anti-VAMP1 antibodies to identify the pre-synaptic compartment (green). Asterisks identify degenerated NMJs while arrows partial innervated ones. Scale bars: 50 μm (10 μm in magnification). Quantification of degenerated, regenerated and partial innervated NMJs in pectoral (B) and intercostal (E) muscles. N = 3, 40 NMJs analyzed/muscle. 2way ANOVA, interaction source of Variation *p < 0,05 in B and ****p <0,0001 in E. C, F) Percentage of NMJ occupancy in pectoral and intercostal muscles, respctively. One way ANOVA **p <0,01, ***p <0,001. (TIF) [file pntd.0011825.s002.tif]
