# Supplementary material for: Agonists of melatonin receptors strongly promote the functional recovery from the neuroparalysis induced by neurotoxic snakes
Source: PLoS Negl Trop Dis. 2024 Jan 8;18(1):e0011825. doi: 10.1371/journal.pntd.0011825 (PMC10798625; doi:10.1371/journal.pntd.0011825)
Supplement: Attachment — Submitted filename: Letter-to-Editor&Reviewers.docx [file pntd.0011825.s004.docx]

Dear Prof Gutierrez,

I thank you for the expert review and for the appreciation of our work which is shared by the three Referees. So much so that they have requested minimal changes that we have introduced in the revised text.

Here following are our replies (in italics) to the comments of the Reviewers (in Arial).

We thank you once more for the expert Editorial handling of this paper and look forward to your reply.

With our best regards

Samuele Negro & Cesare Montecucco

--------------------------------------------------------------------------------------------

**Reviewer #1:**

In this paper, the authors (PNTD-D-23-01512) presented the effects of two drugs, Ramelteon and Agomelatine, licensed for the treatment of insomnia and depression, both agonists of melatonin receptor 1 (MT1), which plays a major role in the recovery of function of the neuromuscular junction (NMJ) after the degeneration of motor axon terminals due to the action of snake venom neurotoxic sPLA2s. The key finding of this work is that the loss of structure and function of the motor neuron axon terminals, after the envenomation of the mouse by the neurotoxic snake (krait) venom, is reversed much more rapidly if treated for at least 4 days with Ramelteon or Agomelatine. The authors propose that these drugs, which are commercially available, safe, non-expensive, have a long bench life and can be administered long after a snakebite even in places far away from health facilities, should be tested on human patients bitten by neurotoxic snakes acting presynaptically for preventing the development of a deadly respiratory paralysis and to speed up their recovery.

The work is meticulously performed and the manuscript clearly written. The results presented in this paper are medically very important.
No modifications are needed. In my opinion, this work should be accepted for publication without a delay.

*We thank very much the Reviewer for the appreciation of our work.*

**Reviewer #2**

In previous work, Montecucco´s group showed that the intercellular signaling axis melatonin-melatonin receptor 1 (MT1) plays a major role in the recovery of function of the NMJs after degeneration of motor axon terminals caused by krait envenomings. Now the authors report on the very promising effect of Ramelteon and Agomelatine, two agonists of MT1 on sell in pharmacies for treatment of insomnia and depression, on the recovery of NMJ function after degeneration of motor axon terminals caused by the venom of Bungarus caeruleus. These drugs cause a relevant recovery of the respiratory function with respect to mice treated with vehicle already after 24 hours from venom injection. Based on these results, the authors suggest that Ramelteon and Agomelatine should be tested for their potential action on the recovery of normal physiology in neuroparalysed patients bitten by neurotoxic snakes.
This is a very interesting investigation with high potential of application in the treatment of envenoming by kraits and other neurotoxic snakes whose venom's mechanism of action is based on nerve terminal damage. I have just a minor comment concerning the dosing of venom and the MT1 agonists:

Mice (25-30 g) "were locally injected in the hind limb with 36 μg/Kg of B. caeruleus venom in 15 μl PBS containing 0.2% gelatin."  Reported LD50 for B. caeruleus is 0.1-0.3 (i.v.)/0.45 (i.m.) μg/g mouse body weight. 36 μg/Kg of B. caeruleus venom of venom equals 0.036 μg/g mouse body weight or 0.08-0.36 LD50s. Right? Why this low dosing?

*The amount of B. caeruleus venom used here is derived from a previous work done with venoms derived from three different species of Bungarus, with two experts of Bungarus venoms (Prof. CH Tan and Dr KY Tan from the Dept. Pharmacology of the University of Malaya)( Stazi M et al An Agonist of the CXCR4 Receptor is therapeutic for the neuroparalysis induced by Bungarus snakes envenoming.* *Clin. Trans. Med. (2022)* [*https://doi.org/10.1002/ctm2.651*](https://doi.org/10.1002/ctm2.651)*.*

*They suggested a range of values and we tested them in preliminary experiments. We were searching for a dose that would paralyse the mice without killing them rapidly, before a regeneration process could take place. This is how the 0.036 μg/Kg value originated. On the other hand, the figure of 0.036  μg/g is not that far from figures reported in the literature ( for example see the LD_50_ value of crude venom of  0.04 mg/kg of mice body weight reported in Int J Pharm Pharm Sci (2014), Vol 7, Issue 1, 239-242, i.v. LD50 values falling within 0.06 to 0.10 μg/g in mice in Journal of Proteomics 164 (2017) 1–18 or the very low value of  LD 50 of 0.02 mg/Kg  found by Prof. CH. Tan, private communication; ).*

On the other hand, "Mice were daily locally injected in the soleus muscle with Ramelteon (29 μg/Kg), diluted in 20 μl PBS containing 0.2% gelatin) or Agomelatine (0.36 mg/Kg). These doses equal 0.8 μg Ramelteon/ μg B. caeruleus venom, 10 μg Agomelatine/ug B. caeruleus. "For Ramelteon we used a dose that corresponds to one fourth of that suggested for long-term use in humans (8 mg/day, taking 70 Kg as average weight), while for Agomelatin we employed the same dose used in humans (25 mg/day)". Dose translation between animal models and human is usually done applying allometric dose scaling (i.e., Reagan-Shaw, S., Nihal, M., Ahmad, N. (2007).
Dose translation from animal to human studies revisited. FASEB J. 22, 659–661; Sharma and McNeill, British Journal of Pharmacology (2009) 157, 907–921; doi:10.1111/j.1476-5381.2009.00267; dosing in mice (mg/Kg) = Dosing in human (mg/Kg) x (Km human/Km mouse). One fourth of 8 mg/70 Kg = 29 μg/Kg (= 0.73-0.87 mg/ 25-30g mouse), whereas allometric translation would suggest mouse (μg/Kg)= 29 μg/Kg * (37/3) = 357.6 μg/Kg = 8.9-10.7 mg (25-30g mouse). Similarly, human dose of 0.36 mg/Kg would correspond to 360 * (37/3) = 4440 μg/Kg = 111-133 mg (25-30g mouse)... Please, comment why linear rather than allometric dose scaling was chosen..

*We thank the Reviewer for raising this highly debated point of doses translation from animal studies to humans. We had initially discussed this point, and have decided to consider the comparison of the amount of body fluids. As this figure is similar in humans and rodents, we decided to take a linear approach. The doses calculated in this way were providing in mice a defined and relevant effect and, accordingly, we continued and performed the planned experiments using the “linear” doses of Ramelteon and Agomelatine.*

*It should also be considered that a appropriate adjustment of drug doses between humans and other vertebrates is almost impossible owing to differences in general physiology, in affinity for receptors, in metabolism and excretion of the drug. Conversion based on the body surface is a reasonable approximation but non devoid of criticisms. It should also be considered that this conversion is nearly always made to determine the animals dose to be used in human trials, whilst, here, we are in the opposite situation. In the end, we will have to suggest the dose to be used for clinical trials of Ramelteon and Agomelatin in human envenomed patients. In the first instance, these doses cannot be different from those already been fixed by FDA and EMA, i.e. 8 mg/Kg of Ramelteon and 25 mg/Kg of Agomelatin per day and we are not intending to change these figures .*

*We would like to thank the Referee for raising this point also because it suggests to us to perform further work of dose and time response in mice.*

**Reviewer #3**

This is a very interesting manuscript reporting the use of ramelteon and agomelatine to combat the paralysis evoked by Bungarus caeruleus. Generally, the paper is well written, but I have a few comments regarding the Methods and how ramelteon and agomelatine were used.

Evoked Junctional Potentials (EJPs) recordings and Compound muscle action potential (CMAP) recordings. How long did it take to inject ramelteon and agomelatine after the snake venom? Were mice maintained anesthetized for 96 h? Explain in detail the general procedure.

*The first injection with agonists Ramelteon or Agomelatinewas made 4 hours after the first injection with Bungarus caeruleus venom.*

*All Bungarus caeruleus venom injections were performed under general anaesthesia via isoflurane inhalation from which mice recover within a few minutes. Instead, daily i.p. injection of agonists does not require anaesthesia. The mice are then anaesthetised and sacrificed after 96 hours, for the analysis of CMAP and EJPs.*

*These informations have been included in the revised text in Material and Methods section in paragraphs Evoked Junction Potential (EJPs) recording and Compound Muscle action potential (CMAP) recordings*

Have you tried to mimic human envenomation and use ramelteon and/or agomelatine after 3-24 after snake venom injection? Do they accelerate the outcome if they are associated with a specific antivenom?

*We expected that this question would have been raised, and we thank the Referee for it.*

*The first injection with Ramelteon or Agomelatine was performed after four hours after the injection of Bungarus caeruleus venom. This time period was chosen because we considered it to be a possible average time from envenoming to first aid. Clearly, a thorough investigation on varying doses of drugs and time of delivery after venom injection is to be performed. Similarly, the use of MT1 agonists before, together or after specific antivenoms has to be tested to mimic what occurs in the real life.*

*We have planned this work and will write soon to ask the National Health Service for the permission of a specific authorization for the animal use. These experiments will then be performed and the results obtained will be the subject of a future publication.*

.
